# The role of Interleukin-10 and 13 in tuberculosis-associated pulmonary dysfunction

**DOI:** 10.22088/cjim.10.2.223

**Published:** 2019

**Authors:** Mohammad Hasan Namaei, Sayyed-gholamreza Mortazavi-Moghaddam, Reza Eslami-Manoochehri, Eslami-Manoochehri Zardast

**Affiliations:** 1 Infectious Diseases Research Center, Birjand University of Medical Sciences, Birjand, Iran; 2 Vali-e-asre Hospital, Birjand University of Medical Sciences, Birjand, Iran

**Keywords:** cytokine, pulmonary function test, pulmonary tuberculosis

## Abstract

**Background:**

Pulmonary dysfunctions are frequently encountered after tuberculosis treatment in clinical practice. In the present study, the role of interleukin-10 and 13 in a tuberculosis-associated pulmonary dysfunction was investigated.

**Methods:**

This is a semi-experimental study on 40 patients selected from referral tuberculosis care center in Birjand, Iran, during 2015-2017. The cases with major medical disorders including those with underlying lung disease were excluded from the study. Informed consent was prepared from each patient, and then blood sample was obtained, serum was extracted and refrigerated at -70 C° at the start (time 1), 2 months (time 2) and 6 months (time 3) after onset of treatment for tuberculosis. Spirometry was also performed at time 2. Finally, 24 patients completed the study.

**Results:**

Of the 24 patients with the mean age of 60.87±21.50 years in the study, 9 (37%) were males and 15 (62.5%) were females. Abnormal spirometry was observed in 20 (83.3%) subjects at time 2, of whom 12 (60%) were restrictive and 8 (40%) obstructive. The mean changes of interleukin 10 from the start to end of the treatment were 89.00±89.47 (P=1.00), -29.36±40.21 (P=0.02) and 3.70±29.98 (P=0.1) in patients with normal, obstructive and restrictive spirometery, respectively (normal vs obstructive and restrictive; p<0.01). While in interleukin 10, changes for interleukin-13 were 77.90±145.97, 6.35±133.10 and -13.35±46.66 (P=0.4), respectively.

**Conclusion:**

Upregulation of IL-10 during tuberculosis treatment might be considered as a protective factor against lung dysfunction. In patients with obstructive form, there was a marked decrease in interleukin-10.

Posttuberculosis pulmonary dysfunction presented as restrictive or obstructive spirometry patterns are frequently reported (1-4). Twenty percent of patients with chronic obstructive pulmonary disease (COPD) have never smoked (5). Inflammatory mechanisms at the origin of infection or autoimmunity play an essential role in COPD pathogenesis (5, 6). In this sense, pulmonary tuberculosis was considered as a risk factor for COPD. The relationship of tuberculosis with COPD was well-described in PLATINO and BOLD studies (7, 8). Cytokines are humoral factors with important role in the regulation of immune response. Interleukin 10 and 13 are classified as Th-2 related cytokines, participating in asthma and COPD pathogenesis. While interleukin 13 is considered as inflammatory promoting, Interleukin 10 has an anti-inflammatory effect and has a low level in patient with asthma and COPD (9-11). Inverse relationship between severity of COPD and serum levels of Interleukin 10 has also been approved (12).

Interleukin 10 level in the bronchoalveolar lavage (BAL) of patients with idiopathic pulmonary fibrosis (fibrotic with little inflammation) is higher than the patients with other ILD diseases with abundant inflammation (13). Conversely, interleukin 13 is known as a profibrotic factor and its biologic targeting could prevent fibrosis (14). Some studies have revealed the changes of interleukin 10 and 13 in relation to pathology of pulmonary tuberculosis. IL-10 gene-disrupted (IL-10 KO) mice get into increased inflammation and injury of lung during pulmonary mycobacterium tuberculosis infection (15). In a reverse order, IL-10 transgenic mice encountered with decreased inflammation, spread of the disease and early death (16). IL-13-over-expressing mice are associated with more necrosis in granuloma and getting more similarity with post primary tuberculosis (17). Interleukin 13 also increases during active tuberculosis (18). Considering the role of interleukin 10 and 13 in pathogenesis of asthma and COPD and also considering occurrence of post tuberculosis pulmonary dysfunction, the role of Interleukin-10 and 13 with respiratory dysfunction during treatment of pulmonary tuberculosis are investigated in the present study

## Methods

This is an experimental study on 40 new adult cases (over 12 years old) of pulmonary tuberculosis selected from referral tuberculosis care center in Birjand, Iran, during 2015-2017, regardless of whether TB is primary or secondary. Informed consent to attendance in the study was obtained from all patients. Patients with any underlying disease other than pulmonary tuberculosis and also smokers were excluded from the study. The diagnostic criteria were initially based on clinical and radiologic evidence compatible with pulmonary tuberculosis in the presence of one of the following conditions. Definitive diagnosis was confirmed by culture of sputum or BAL for mycobacterium tuberculosis (MTB) in all cases.

1. Two samples of sputum smear positive for MTB
2. One sample of sputum smear and one sputum culture positive for MTB
3. One sample of BAL smear and one BAL culture positive for MTB
4. One sample of BAL or sputum smear/culture positive for MTB in patients with radiological and clinical findings strongly suggestive of pulmonary tuberculosis

Blood sample (5ml) was obtained, serum extracted and refrigerated at -70 C° from eligible patients at times of start (time 1), 2 months (time 2) and 6 months(time 3) after onset of treatment for pulmonary tuberculosis. All patients were treated free of charge based on WHO (World Health Organization) protocols for tuberculosis treatment by the referral tuberculosis care center in Birjand, Iran. Every two months, examinations for all patients were done by the pulmonologist during the course of tuberculosis treatment. Treatment response was confirmed at the end of treatment by clinical findings and study of chest radiography, smear and culture of sputum or BAL samples. IL-10 and IL-13 measurements were performed by ELISA method using the INTEGRA 400 device and the Roche Diagnostics Kit, Germany and reported quantitatively. Inter and intra assay coefficient of variation respectively for both interleukin 10 and 13 was <12% and <10%. Spirometry was performed at time 2. Data are recorded and analyzed using SPSS software (Version 23). Shapiro-Wilk test was used to evaluate the normality of the data. Mann-Whitney and Kruskal-Wallis tests were used to compare means in 2 and 3 independent groups with non-normal distribution. We used Friedman test to compare the mean of repeated measures with non-normal distribution. The approval number of the study was IR.BUMS.REC.1395.70 recorded in the Ethics Committee of Birjand University of Medical Sciences, Birjand, Iran. 

## Results

From 40 recruited patients, 24 cases with the mean age of 60.87±21.50 years completed the study. Out of 24 cases, 9 (37.5%) were men and 15(62.5%) were women. Patterns of spirometry were normal, restrictive and obstructive in 4(16.7%), 12(50%) and 8(33.3%) cases, respectively. The mean reduction of IL-10 during treatment in TB patients with obstructive spirometry was -29.36±40.21 (P=0.02). On the contrary, IL-10 was set up in patients with normal spirometry (89.07±89.47, P=0.1) (table 1). Mean changes in IL-10 and 13 levels from start to end of study (time1 to time3) showed a strong negative (r=-0.72, P=0.04), a weak negative (-0.08 (0.79), and a strong positive (0.87 (0.12) relationships in patients with obstructive, restrictive and normal spirometry respectively (table 2). There were also significant relationships between IL-10 and IL-13 in patients with obstructive spirometry at times 2 (r= 0.81, P=0.01) and 3 (r= 0.76, P=0.02) (table 2). 

**Table1:** Mean comparison of interleukin 10 and 13 between and within spirometry pattern groups alongside of TB treatment

**Interleukin**	**Restrictive(N=12)** **(Mean±SD)**	**Obstructive( N=8)** **(Mean±SD)**	**Normal (N=4)** **(Mean±SD)**	**P value**
Interleukin -10 (time1)	85.13±43.71	124.70±91.41	79.52±49.35	0.15
Interleukin -10 (time2)	91.11±65.25	111.06±103.90	100.05±55.37	0.85
Interleukin -10 (time3) P value(within group) Mean changes	88.83±66.34 1.00 3.70±29.98	95.33±61.51 0.02 -29.36±40.21	168.60±137.15 0.10 89.07±89.47	0.18 0.00*
Interleukin -13 (time1) Interleukin -13 (time2) Interleukin -13(time3) P value(within group) Mean changes	115.98±49.92 105.50±29.62 102.62±34.97 0.26 -13.35±46.66	138.72±53.42 138.26±108.73 145.07±107.74 0.22 6.35±133.10	126.52±62.07 128.32±32.90 204.42±185.68 1.00 77.90±145.97	0.67 0.37 0.31 0.4

time1: initiation of TB treatment, time 2: at two months after starting TB treatment, time 3: at end of 6 months TB therapy

* : Normal versus restrictive and obstructive with statistically significant different

**Table 2:** Relationship between changes of IL-10 and 13 in all patients, and patients with normal, restrictive and obstructive spirometry

**Time of interleukin test**	**Interleukin-10**	**Interleukin-13**	**Correlation (P-value)**
***All patients***			
Time 1	97.38±64.56	125.32±51.68	0.3 (0.14)*
Time 2	99.25±76.19	120.22±66.31	0.63 (0.00)*
Time3	104.29±81.13	133.74±100.17	0.75 (0.00)*
Mean changes(from time1 to 3)	6.90±60.03	8.42±101.49	0.19 (0.35)*
***Normal spirometry group(N=4)***			
Time 1	79.52±49.35	126.52±62.07	0.88 (0.11)#
Time 2	100.05±55.37	128.32±32.90	0.32 (0.67)≠
Time 3	168.60±137.15	204.42±185.68	0.92 (0.07)≠
Mean changes(from time1 to 3)	89.07±89.47	77.90±145.97	0.87 (0.12)≠
***Restrictive spirometry group( N=12)***			
Time 1	85.13±43.71	115.98±49.92	0.15 (0.63)*
Time 2	91.11±65.25	105.50±29.62	0.68 (0.04)*
Time 3	88.83±66.34	102.62±34.97	0.72 (0.00)*
Mean changes(from time1 to 3)	3.70±29.98	-13.35±46.66	-0.08 (0.79)*
***Obstructive spirometry group(N=8)***			
Time 1	124.70±91.41	138.72±53.42	-0.15 (0.72)≠
Time 2	111.06±103.90	138.26±108.73	0.81 (0.01)*
Time 3	95.33±61.51	145.07±107.74	0.76 (0.02)*
Mean changes(from time1 to 3)	-29.26±40.21	6.35±133.10	-0.72 (0.04)≠

* Spearman's rho test,

≠ Pearson, time1: initiation of TB treatment, time 2: at the end of 2 months TB treatment, time 3: at the end of 6 months TB therapy

## Discussion

The prevalence of abnormal spirometry was 83.3% in our study 2 months after starting pulmonary tuberculosis treatment. Meanwhile, the prevalence of post tuberculosis abnormal spirometry reported 74% in Tanzania and 59% in America (19, 20). Taken together these studies mean that lung dysfunction appears and progress alongside with pulmonary tuberculosis regardless of antibacterial treatment. The frequency of restrictive, obstructive and normal spirometry was 50%, 33.3% and 16.7% respectively in our study. However, reported frequency of restrictive, obstructive and mixed type of spirometry were respectively 31%, 15% and 13% in America (20), 13%, 42% and 19% in Tanzania (19) 29.7%, 55.3%, and 14.8% in Pakistan (21). More prevalence of restrictive spirometry in our study may be due to performing spirometry throughout the course during instead of post tuberculosis treatment. Scientific scholars have much emphasis on the development of post tuberculosis chronic obstructive pulmonary disease and pulmonary tuberculosis has been considered as a major risk factor for COPD by GOLD (2016) (22).

Serum levels of IL-10 showed significant upregulation in patients with normal and downregulation with obstructive spirometry alongside tuberculosis treatment in our study. IL-10 is known as an anti-inflamatory cytokine and its production in patients with pulmonary infection leads to less inflammation-related lung damages (23). Since tuberculosis is considered as a risk factor for COPD (24), change in IL-10 can be raised to the development of post tuberculosis lung dysfunction (9-11, 25). Patients with restrictive spirometry did not show significant change in IL-10 during tuberculosis treatment in our study. The finding can be in consistent with the assumption that high levels of IL-10 can help in preventing post infectious diseases tissue damage (26). Interleukin 10 can also be considered as an anti-inflammatory drug in preventing pulmonary fibrosis in patients with idiopathic pulmonary fibrosis (IPF) (27).

Changes in IL-13 were not significant during tuberculosis treatment in patients with normal, restrictive or obstructive spirometry in our study. There is not much information about the role of interleukin 13 in patients with tuberculosis and also post tuberculosis complications. It was claimed in a study that expression of IL-13 was upregulated in Indian children with latent TB compared with that in controls (28). Despite claimed role of IL-13 in pathogenesis of tuberculosis, its relationship with respiratory dysfunction is not clear. The role of IL-13 in respiratory dysfunction is even more questioned in patients with COPD and asthma in a study conducted by Grubek-Jaworska H et al. (29). Patients with obstructive spirometry showed inverse relationship between changes of IL-10 and IL-13 from start to end of therapy. The positive relationship, however, is observed in patients with normal spirometry. Other studies have also shown that interleukin 10 and 13 have an inverse relationship in patients with COPD and asthma (9-11). Based on significant reduction of IL-10 in tuberculosis patients with obstructive spirometry in our study, it can be concluded that IL-10 plays a central role in preventing respiratory dysfunction. This assumption was also confirmed by a study that explained more severe COPD in condition with low levels of IL-10 (12). Limitations of the study: The most important limitation in our study was that of not-having spirometry before starting TB treatment. To minimize these effects, the patients with other risk factors for COPD were excluded from the study. Low number of participants was another limitation of this study. 

Conclusion: Respiratory dysfunction as a post pulmonary tuberculosis complication is common. IL-10 and IL-13 are two cytokines that undergo changes in the course of tuberculosis and can be raised in pathogenesis of lung dysfunction. It seems that the low levels of interleukin 10 play an essential role in the emergence of tuberculosis related obstructive and also restrictive pulmonary dysfunction. 

## References

[1] Shah M, Reed C (2014). Complications of tuberculosis. Curr Opin Infect Dis.

[2] Amaral AF, Coton S, Kato B (2015). Tuberculosis associates with both airflow obstruction and low lung function: BOLD results. Eur Respir J.

[3] Chushkin MI, Ots ON (2017). Impaired pulmonary function after treatment for tuberculosis: the end of the disease?. J Bras Pneumol.

[4] Hnizdo E, Singh T, Churchyard G (2000). Chronic pulmonary function impairment caused by initial and recurrent pulmonary tuberculosis following treatment. Thorax.

[5] Lamprecht B, McBurnie MA, Vollmer WM (2011). COPD in never smokers: results from the population-based burden of obstructive lung disease study. Chest.

[6] Cosio MG, Saetta M, Agusti A (2009). Immunologic aspects of chronic obstructive pulmonary disease. N Engl J Med.

[7] Perez-Padilla R, Fernandez R, Lopez Varela MV (2012). Airflow obstruction in never smokers in five Latin American cities: the PLATINO study. Arch Med Res.

[8] Buist AS, Vollmer WM, Sullivan SD (2005). The Burden of Obstructive Lung Disease Initiative (BOLD): rationale and design. COPD.

[9] Berger A (2000). Th1 and Th2 responses: what are they?. BMJ.

[10] Gong Y, Shi GC, Wan HY (2013). Association between the interleukin-13 gene and development of chronic obstructive pulmonary disease in southern Chinese Han population: a case-control study. Chin Med J (Engl).

[11] Corren J (2013). Role of interleukin-13 in asthma. Curr Allergy Asthma Rep.

[12] Silva BSA, Lira FS, Ramos D (2018). Severity of COPD and its relationship with IL-10. Cytokine.

[13] Kucejko W, Chyczewska E, Naumnik W, Ossolińska M (2009). Concentration of surfactant protein D, Clara cell protein CC-16 and IL-10 in bronchoalveolar lavage (BAL) in patients with sarcoidosis, hypersensivity pneumonitis and idiopathic pulmonary fibrosis. Folia Histochem Cytobiol.

[14] Murray LA, Zhang H, Oak SR (2014). Targeting interleukin-13 with tralokinumab attenuates lung fibrosis and epithelial damage in a humanized SCID idiopathic pulmonary fibrosis model. Am J Respir Cell Mol Biol.

[15] Higgins DM, Sanchez-Campillo J, Rosas-Taraco AG (2009). Lack of IL-10 alters inflammatory and immune responses during pulmonary Mycobacterium tuberculosis infection. Tuberculosis (Edinb).

[16] Schreiber T, Ehlers S, Heitmann L (2009). Autocrine IL-10 induces hallmarks of alternative activation in macrophages and suppresses antituberculosis effector mechanisms without compromising T cell immunity. J Immunol.

[17] Heitmann L, Abad Dar M, Schreiber T, Erdmann H (2014). The IL-13/IL-4Rα axis is involved in tuberculosis-associated pathology. J Pathol.

[18] Jeong YH, Hur YG, Lee H, Kim S (2015). Discrimination between active and latent tuberculosis based on ratio of antigen-specific to mitogen-induced IP-10 production. J Clin Microbiol.

[19] Manji M, Shayo G, Mamuya S (2016). Lung functions among patients with pulmonary tuberculosis in Dar es Salaam - a cross-sectional study. BMC Pulm Me.

[20] Pasipanodya JG, Miller TL, Vecino M (2007). Pulmonary impairment after tuberculosis. Chest.

[21] Baig IM, Saeed W, Khalil KF (2010). Post-tuberculous chronic obstructive pulmonary disease. J Coll Physicians Surg Pak.

[22] King PT (2015). Inflammation in chronic obstructive pulmonary disease and its role in cardiovascular disease and lung cancer. Clin Transl Med.

[23] Kumar A, Creery WD (2000). The therapeutic potential of interleukin 10 in infection and inflammation. Arch Immunol Ther Exp (Warsz).

[24] Sarkar M, Srinivasa, Madabhavi I, Kumar K (2017). Tuberculosis associated chronic obstructive pulmonary disease. Clin Respir J.

[25] Chung F (2001). Anti-inflammatory cytokines in asthma and allergy: interleukin-10, interleukin-12, interferon-gamma. Mediators Inflamm.

[26] Redford PS, Murray PJ, O'Garra A (2011). The role of IL-10 in immune regulation during M tuberculosis infection. Mucosal Immunol.

[27] Millar AB (2006). IL-10: Another therapeutic target in idiopathic pulmonary fibrosis?. Thorax.

[28] Dhanasekaran S, Jenum S, Stavrum R (2013). Identification of biomarkers for Mycobacterium tuberculosis infection and disease in BCG-vaccinated young children in Southern India. Genes Immun.

[29] Grubek-Jaworska H, Paplińska M, Hermanowicz-Salamon J (2012). IL-6 and IL-13 in induced sputum of COPD and asthma patients: correlation with respiratory tests. Respiration.

